# Japanese preference weights of the Adult Social Care Outcomes Toolkit for Carers (ASCOT-Carer)

**DOI:** 10.1007/s11136-021-03076-w

**Published:** 2022-01-12

**Authors:** Takeru Shiroiwa, Hiromi Nakamura-Thomas, Mai Yamaguchi, Mie Morikawa, Yoko Moriyama, Takashi Fukuda, Stephen Allan, Juliette Malley

**Affiliations:** 1 Center for Outcomes Research and Economic Evaluation for Health (C2H), National Institute of Public Health, 2-3-6 Minami, Wako, Saitama 351-0197 Japan; 2 Graduate School of Health, Medicine and Welfare, School of Occupational Therapy, Saitama Prefectural University, 820 Sannomiya, Koshigaya, Saitama 343-8540 Japan; 3 Japan Lutheran College, 3-10-20 Osawa, Mitaka, Tokyo 181-0015 Japan; 4 Department of Policy Studies, Tsuda University, 1-18-24 Sendagaya, Shibuya-ku, Tokyo, 151-0051 Japan; 5 Department of Health and Welfare Services, National Institute of Public Health, 2-3-6 Minami, Wako, Saitama 351-0197 Japan; 6 Personal Social Services Research Unit (PSSRU), University of Kent, Canterbury, CT2 7NF Kent UK; 7 Care Policy and Evaluation Centre (CPEC), London School of Economics and Political Science, Houghton Street, London, WC2A 2AE UK

**Keywords:** ASCOT, Preference, Best–worst scaling (BWS), Time trade-off (TTO), Quality of life, Caregiver

## Abstract

**Purpose:**

We developed preference weights of the Adult Social Care Outcomes Toolkit for Carers (ASCOT-Carer) in Japan.

**Methods:**

We used best–worst scaling (BWS) and composite time trade-off (cTTO) to determine the preference weights for ASCOT-Carer states in the general population. TTO values were applied to convert the BWS scores to utilities. The sample number was approximately 1000 for the BWS survey and 200 for the TTO survey. Whereas face-to-face surveys by computer-assisted interviewing were adopted for the TTO tasks, a web-based survey was used for the BWS tasks. In the BWS tasks, the ASCOT-Carer states were presented, and the “best,” “worst,” “second best,” and “second worst” domains in a profile were selected. A mixed logit model was applied to the BWS data.

**Results:**

The respondents’ background was similar to that of the general population, although the number of people in the age and sex categories was equal. The preference weights for calculating the utilities of the ASCOT-Carer states were estimated. The estimated utilities of the ASCOT-Carer states were distributed between 1 and 0.02. All preference weights were consistent. The item with the highest preference weight was level 1 in the “space and time to be yourself.” The least preferred item was level 4 in the “space and time to be yourself” and “control over daily life” domains.

**Conclusion:**

We established Japanese preference weights for ASCOT-Carer states, the first weights of an Asian country. The estimated utilities can contribute to the measurement of caregivers’ social care-related QoL and perform of cost-effectiveness analyses.

**Supplementary Information:**

The online version contains supplementary material available at 10.1007/s11136-021-03076-w.

## Introduction

Many developed countries, such as Japan, are experiencing rapid growth in the size and proportion of older persons in their population. In the year 2020, the Organization for Economic Co-operation and Development (OECD) reported that the top 3 countries with the largest proportion of elderly people (aged 65 years and over) are Japan with 28.7%, Italy with 23.3%, and Portugal with 22.8% [1]. As the rate of aging in populations is increasing, the roles of informal caregivers are also increasing. For example, in Japan, the main caregivers in about 68% of the cases are family members of care recipients [2].

Under these circumstances, it is important to establish an instrument to evaluate informal caregivers’ quality of life (QoL). Preference-based measures (PBMs) for informal caregivers, which can be used to calculate quality-adjusted life years (QALYs), are limited, except for CareQol [3] and the Carer Experience Scale (CES) [4]. The economic evaluation of a care program is important when considering the efficiency of the intervention. Therefore, a research group at the University of Kent, the United Kingdom, developed the Adult Social Care Outcomes Toolkit for caregivers (ASCOT-Carer), [5, 6] which is designed to measure the utility of informal caregivers. Originally, the SCT4 version of ASCOT was developed [7, 8] which is for care receivers, not caregivers. The ASCOT SCT4 consists of the following eight domains: control over daily life, personal cleanliness and comfort, food and drink, personal safety, social participation and involvement, occupation, accommodation cleanliness and comfort, and dignity; three of these domains (control over daily life, social participation and involvement, and occupation) overlap with the ASCOT-Carer. Our research group developed a Japanese version of the ASCOT four-level self-completion questionnaire (SCT4) [9] and value sets for ASCOT SCT4 [10]. Translation of ASCOT-Carer to Japanese was also completed.

In the area of health technology assessment (HTA), the National Institute for Health and Care Excellence (NICE) in the UK clarified the focus of the outcomes as “all direct health effects, whether on the patients, or when relevant, carers.” [11] The QoL of informal caregivers may influence the recommendations, but in practice, few evaluations consider the utility of caregivers [12]. The situation is the same with academic papers on economic evaluation [13, 14].

Given these considerations, we developed Japanese preference weights for ASCOT-Carers. ASCOT-Carer preference weights have already been developed in the UK [15] and Austria [16]. This is the first report of preference weights for an Asian country and the first survey to evaluate caregivers’ health state by time trade-off (TTO). Cultural differences between the UK and Japan could potentially influence caregivers’ health-related QoL and preference weights; therefore, it is important to develop new weights for Japanese ASCOT-Carers to reflect Japanese peoples’ general preferences. We also explored the differences in preference weights between the two countries.

## Method

### ASCOT-Carer

The Japanese version of ASCOT-Carer [6] was used in this study, with permission from and in collaboration with the developer of the original measure—the ASCOT team of the Personal Social Services Research Unit (PSSRU) at the University of Kent. The ASCOT-Carer consists of seven domains: (1) occupation, (2) control over daily life, (3) looking after oneself, (4) personal safety, (5) social participation and involvement, (6) space and time to be yourself, and (7) feeling supported and encouraged. Each domain is represented by one item and has four response options. The first level among the four responses indicates the best health state, and the fourth level indicates the worst health state.

### Best–worst scaling and time trade-off

We used best–worst scaling (BWS) and composite time trade-off (cTTO) to measure the preference weights of ASCOT-Carer states in the general population. The TTO values were applied to convert the BWS scores to utility values. In the UK survey, the cTTO values were not measured, and the BWS scores were converted by assuming the utility value of the worst state as zero. In this study, we showed those taking part in the TTO survey a profile including each ASCOT-Carer domain. Respondents were asked to put themselves in an imaginary state (described by the profile) of being caregivers and then select the best, worst, second best, and second worst domains from the profile. The selected domains were greyed out on the screen, and the remaining domains were presented for the next choice. In the BWS phase, four blocks consisting of eight ASCOT-Carer profiles were randomly allocated to each respondent; 32 profiles were selected from all 4^8^ = 65,536 profiles using a fractional–factorial design.

In the TTO survey, participants always started with a conventional TTO task assuming that they were informal caregivers: living for 10 years in a health state described by the ASCOT-Carer, or living x years in full health. If the participants considered the presented ASCOT-Carer state to be better than immediate death (i.e., *x* > 0), the value of *x* was varied until indifference was reached. If the participants considered immediate death to be better than living for 10 years in the ASCOT-Carer state (i.e., *x* < 0), a lead-time TTO [17, 18] was started that allowed estimation of negative values. In lead-time TTO, a series of choices were offered between “*y* years of life in full health” and a life of “10 years in full health followed by 10 years in the presented ASCOT-Carer state.” The value of *y* was varied until indifference was reached. In the cTTO phase, four blocks were similarly allocated to each respondent. Each block consisted of eight ASCOT states. However, only the worst states [4444444] were included in the two blocks. In total, 31 ASCOT-Carer profiles (= 4 × 8 − 1) were used for the TTO survey.

### Subjects and the survey process

An online survey was conducted during the BWS phase. Respondents (aged 20 to 79 years) were recruited through a Japanese web panel based on quota sampling by sex and age. The sample size was 1000. The Japanese sample number was not based on any statistical considerations but was selected with reference to a UK survey [15].

First, the respondents self-assessed their own QoL using the ASCOT-Carer. Then, the respondent was asked to value the eight ASCOT-Carer profiles based on the BWS. Each domain in a profile is shown line by line. The position of each domain was randomized between the people to avoid positioning effects. The order in which the eight health states in the block were presented was randomized. After the BWS tasks were completed, the degree of understanding of the BWS questions, experience with social care, and demographic data were collected from the respondents. The response times of the BWS tasks were recorded.

For the TTO, face-to-face surveys were performed because the TTO tasks are more complex, and it was considered that a web-based survey might create some biases [19]. The subjects were different from the respondents in the BWS tasks. The respondents (aged 20 to 64 years) were recruited through a panel owned by a research company based on non-random quota sampling by sex and age. As it was difficult to recruit elderly people for this survey because of the COVID-19 outbreak, respondents aged more than 65 years were not included. The inclusion criteria were as follows: (1) aged 20 to 65 years; (2) current Japanese residency; (3) ability to visit the survey room in Tokyo; (4) ability to provide informed consent; and (5) ability to complete the tasks in Japanese. The target sample number was approximately 200. This was not based on statistical considerations, but 50 responses per health state were collected. Respondents were asked to visit a survey center in Tokyo. Computer-assisted personal interviewing (CAPI) was performed with interviewers’ support, with a one-on-one setting over intervals of 30 to 60 min at the survey center. Subsequently, three training TTO tasks were completed before the actual TTO tasks; “in a wheelchair,” “much better than being in a wheelchair,” and “much worse than being in a wheelchair, so bad that one would prefer to die immediately” Are the responses that were collected automatically as electronic data.

The survey was conducted from January 2021 to March 2021. Prior to conducting the survey, the investigators at each location received training for approximately half a day. Screenshots of the BWS and TTO surveys are provided in the Appendix.

### Statistical analysis

A mixed logit model (MIXL) [20] was used to analyze the BWS data. The best, worst, second best, and second worst data were pooled for the analysis. A mixed logit model can analyze the heterogeneity of coefficients by relaxing the assumption of independence of irrelevant alternatives (IIA), whereas a simple multinomial logit (MNL) model assumes that all responses are independent.

Mixed logit models include 7 (domain level) + 3 × 8 − 1 (item level; level 1 to 3 in each domain excluding the reference level: the third level of “space and time to be yourself”) = 30 dummy variables to be estimated. When choices are analyzed based on random utility theory, *U*_*ij*_ (utility respondent *j* derives from choosing item *i*) is divided into an explainable component (*V*_*ij*_) and a random component (*ε*_*ij*_).

$$U_{ij} = V_{ij} + \, \varepsilon_{ij}$$

$$V_{ij} = \beta_{1} X_{1} + \, \beta_{2} X_{2} + \cdots + \beta_{7} X_{7} + \, \beta_{11} X_{11} + \, \beta_{12} X_{12} + \cdots + \beta_{73} X_{73} ,$$

where *β*_p_ denotes the common effect of the pth ASCOT domain, and *β*_pq_ denotes the effect of the qth (1 ≤ *q* ≤ 3) level of the pth domain. *β*_p_ are random parameters and *β*_pq_ are fixed parameters. In the mixed logit model, if *β*_p_^m^ and *β*_p_^s^ are used to represent the mean and scale parameters, respectively, for the random coefficient *β*_p_,

$$\beta_{p} = \beta_{p}^{m} + \beta_{p}^{s} \cdot \eta$$

where *η* is a stochastic component with normal distribution.

*X*_*i*_ and *X*_*ij*_ indicate the choice of information. For example, if respondents selected the first level of the second domain as the best item, *X*_2_ = 1, *X*_21_ = 1, and others = 0. In the case of the worst and second worst choices, − 1 was used instead of 1 [27] parameters in the utility function were estimated with *mixlogit* in STATA 16. Respondents with a total BWS time of < 4.5 min, which was considered too short based on the pre-test results of a valuation survey in the UK, were excluded.

Regarding the TTO data, when the respondents equated 10 years of life with a better-than-dead ASCOT-Carer state to *x* years of life in perfect health, the TTO value was calculated as *x*/10*.* Conversely, when y years of life with a perfect ASCOT-Carer state was equated to “life with a perfect ASCOT-Carer state for 10 years, followed by life with a worse-than-dead ASCOT-Carer state for 10 years,” then the TTO value was calculated as *y*/10 − 1. Summary statistics of the TTO values of the 31 ASCOT-Carer states were calculated.

Finally, to convert the latent BWS scores to utility values, the function *f*(∙) between the latent BWS scores and TTO values of the 31 ASCOT-Carer states was estimated as TTO_*i*_ = *f* (*BWS*_*i*_) + *ε*_*i*_, where TTO_i_ denotes the observed mean TTO value, and BWS_i_ denotes the latent BWS score for the *i*th ASCOT-Carer state (1 ≤ *i* ≤ 31).

## Results

The collected samples included 1115 respondents for the BWS tasks (914 respondents with a total BWS time of ≥ 4.5 min were included in the analysis) and 220 participants for the TTO tasks. The mean and median total response times of 914 respondents to the BWS questions were 10.1 min (standard deviation (SD): 6.1) and 8.5 min (interquartile range (IQR) 6.3–11.8 min), respectively, if people with response times of greater than 60 min were excluded from this calculation. Appendix incudes the comparison of respondents’ backgrounds between those included in the analysis (*N* = 914) and excluded from the analysis (*N* = 201).

Regarding the degree of understanding of the BWS tasks, only 71 (7.8%) of the respondents reported that they could not imagine the presented ASCOT-Carer states and the differences among the eight states. The difficulty levels of the BWS tasks were “very easy” (3.6%), “quite easy” (8.8%), “slightly easy” (26.4%), “slightly difficult” (49.1%), “quite difficult” (9.7%), and “very difficult” (3.4%). A total of 91.8% could compare the seven domains included in each profile.

In the case of the TTO tasks, the mean and median total response times to the TTO questions were 19.9 min (SD: 5.3) and 19.4 min (IQR 16.1–22.5 min), respectively; 8.2% could imagine the described ASCOT-Carer states very easily, 35.5% quite easily, 50.5% with some difficultly, and 5.9% with great difficulty. The difficulty levels of the TTO tasks were “very easy” in 11.8%, “quite easy” in 35.5%, “quite difficult” in 41.8%, and “very difficult” in 10.9% of cases.

### Demographic factors

The respondents’ background characteristics in the BWS and TTO populations are shown in Table 1. The median household income of the BWS population ranged from JPY 5 million to JPY 7 million. When compared with the household incomes of all Japanese families of JPY 4.4 million in 2019, [2] the household income of the BWS population was high. According to the 2019 Labour Force Survey, [28] full-time workers accounted for 31.6% of all workers, and part-time workers accounted for 13.7%. Of the total, 24.3% of Japanese individuals graduated from a university or graduate school in 2017; 61.3% of the Japanese people were married and 31.6% were unmarried in 2015. Many factors (excluding the age category) were comparable with observations in the general population.

**Table 1:** Demographic factors of respondents

	BWS population (*N* = 914)		TTO population (*N* = 220)	
*Gender*				
Male	435	47.6%	110	50.0%
Female	479	52.4%	110	50.0%
*Age*				
20–29	121	13.2%	44	20.0%
30–39	130	14.2%	44	20.0%
40–49	135	14.8%	44	20.0%
50–59	163	17.8%	44	20.0%
60–69	180	19.7%	44	20.0%
70–79	185	20.2%	–	–
*Population of living municipality*				
> 1,500,000	232	25.4%	–	–
500,000–1,500,000	176	19.3%	–	–
200,000–500,000	188	20.6%	–	–
50,000–200,000	217	23.7%	–	–
< 50,000	101	11.1%	–	–
*Employment*				
Full-time worker	287	31.4%	134	60.9%
Part-time worker	133	14.6%	35	15.9%
Self-employment	76	8.3%	9	4.1%
Retired	97	10.6%	3	1.4%
Houseworker	181	19.8%	18	8.2%
Student	32	3.5%	18	8.2%
Others	108	11.8%	3	1.4%
*Marital status*				
Unmarried	293	32.1%	83	37.7%
Married	536	58.6%	125	56.8%
Others	85	9.3%	12	5.5%
*Education*				
Elementary or Junior high school	27	3.0%	0	0.0%
High school	275	30.1%	49	22.3%
College	182	19.9%	60	27.3%
University or graduate	424	46.4%	111	50.5%
Others	6	0.7%	0	0.0%
*Household income (JPY)*				
< 1 million	44	4.8%	1	4.5%
1 million–2 million	68	7.4%	3	1.4%
2 million–3 million	94	10.3%	13	5.9%
3 million–4 million	120	13.1%	25	11.4%
4 million–5 million	116	12.7%	23	10.5%
5 million–7 million	133	14.6%	52	23.6%
7 million–10 million	122	13.4%	49	22.3%
10 million–15 million	51	5.6%	26	11.8%
15 million–20 million	12	1.3%	8	3.6%
> 20 million	7	0.8%	4	1.8%
Unknown	147	16.1%	16	7.3%

### Results of the BWS and TTO tasks

**Fig. 1:**
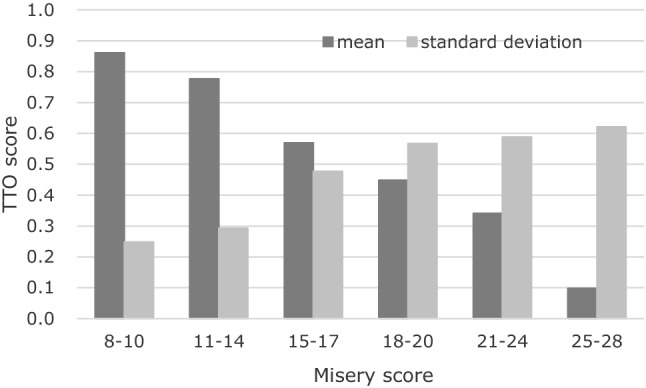
Misery score and mean and standard deviation of the TTO values

**Fig. 2:**
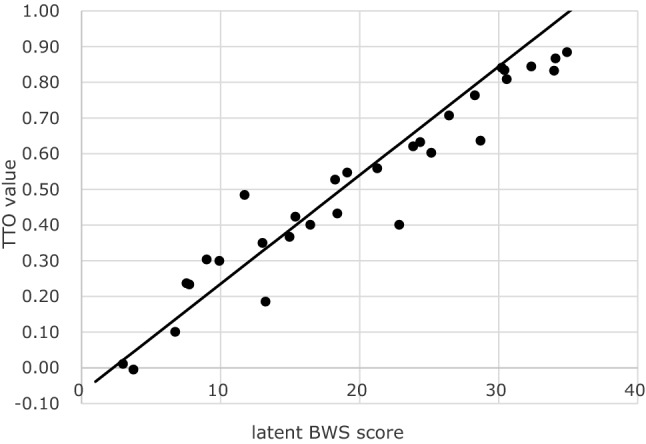
Relationship between the latent BWS score and TTO values

Table 2 presents the estimated coefficients using the mixed logit model. The fourth level of “space and time to be yourself” was used as the reference, similar to that in the UK model, and all the coefficients were positive. Table 3 presents the mean TTO values of ASCOT-Carer states. The worst TTO value was 0.011 [4444444], and the best value was 0.867 [1112121]. Only one health state, [4344444], was evaluated as “worse than death” (− 0.01); however, the absolute value was small. We confirmed the face validity of the TTO survey, as shown in Fig. 1. As the misery score (the sum of the level scores across all dimensions) increased, the mean TTO value decreased, and the standard deviation increased as the misery score increased. Figure 2 shows the relationships between the latent BWS scores and TTO values of the 31 states. Based on the linear relationship between latent BWS scores and TTO values, the BWS scores can be converted to utility values using the following formula:

1 $${\text{TTO}}\;{\text{value}} = 0.0305*{\text{BWS}}\;{\text{score }} - 0.0695$$

(*R*^2^ = 0.98 and mean square error = 0.09).

**Table 2:** Results of the BWS survey (*N* = 914)

Variables	Domain	Coefficient	SE	*P*-value
*β*_1_	Occupation	0.908	0.060	< 0.001
SD(*β*_1_)		0.000	0.227	
*β*_2_	Control over daily life	0.292	0.060	< 0.001
SD(*β*_2_)		0.118	1.295	
*β*_3_	Looking after yourself	0.666	0.061	< 0.001
SD(*β*_3_)		0.741	0.152	
*β*_4_	Personal safety	0.400	0.060	< 0.001
SD(*β*_4_)		0.834	0.150	
*β*_5_	Social participation and involvement	0.346	0.060	< 0.001
SD(*β*_5_)		0.975	0.135	
*β*_6_	Space and time to be yourself	2.062	0.064	< 0.001
SD(*β*_6_)		0.509	0.138	
*β*_7_	Feeling supported and encouraged	0.360	0.068	< 0.001
SD(*β*_7_)		1.114	0.124	
*β*_11_	Occupation	4.764	0.067	< 0.001
*β*_12_		4.549	0.062	< 0.001
*β*_13_		1.249	0.062	< 0.001
*β*_21_	Control over daily life	4.532	0.066	< 0.001
*β*_22_		4.085	0.065	< 0.001
*β*_23_		0.756	0.060	< 0.001
*β*_31_	Looking after yourself	4.703	0.068	< 0.001
*β*_32_		4.433	0.066	< 0.001
*β*_33_		0.952	0.060	< 0.001
*β*_41_	Personal safety	3.891	0.067	< 0.001
*β*_42_		3.237	0.067	< 0.001
*β*_43_		1.118	0.059	< 0.001
*β*_51_	Social participation and involvement	3.612	0.070	< 0.001
*β*_52_		3.038	0.070	< 0.001
*β*_53_		2.143	0.063	< 0.001
*β*_61_	Space and time to be yourself	4.683	0.071	< 0.001
*β*_62_		4.301	0.071	< 0.001
*β*_71_	Feeling supported and encouraged	3.904	0.079	< 0.001
*β*_72_		3.485	0.077	< 0.001
*β*_73_		1.757	0.067	< 0.001

Number of respondents = 914

Number of observations = 160,864

Number of cases = 29,248

Log simulated-likelihood = − 33,317.084

Wald *chi*^2^(27) = 12,557.61, Prob > *chi*^2^ < 0.001

*SD* standard deviation of random coefficient

**Table 3:** TTO values of the 31 ASCOT-Carer states

State	*N*	Mean
1112121	55	0.867
1131122	55	0.809
1131221	55	0.835
1144222	55	0.603
1241432	55	0.559
1314443	55	0.367
1322113	55	0.764
2111111	55	0.885
2122111	55	0.833
2124424	55	0.401
2141111	55	0.841
2221321	55	0.845
2234134	55	0.527
2431322	55	0.633
2433111	55	0.621
2444334	55	0.485
3133342	55	0.401
3324333	55	0.424
3343433	55	0.300
3344443	55	0.101
3421121	55	0.707
3434433	55	0.304
4131341	55	0.433
4212211	55	0.636
4332234	55	0.350
4333221	55	0.547
4344433	55	0.237
4344444	55	− 0.005
4434433	55	0.234
4444323	55	0.185
4444444	110	0.011

### Preference weight

The preference weights for calculating the latent BWS scores of the ASCOT-Carer states listed in Table 4. These values were calculated using the BWS coefficients listed in Table 2 and Eq. (1). All the coefficients in Table 4 were consistent; weights at the higher level in the same domain were higher, and those at the lower level were lower. By adding the weight of each domain, the utility value of the ASCOT-Carer states can be calculated. For example, in the case that a response to ASCOT-Carer was [2234134,] the utility can be calculated using Table 4 as follows: 0.166 + 0.133 + 0.049 + 0.012 + 0.121 + 0.063 + 0.011 − 0.069 (intercept: always need to include except full QoL status) = 0.486. The utility values of the ASCOT-Carer states were distributed between 1.00 and 0.02. The estimated utility value of the worst states by UK weighting was the same at − 0.001.

**Table 4:** Japanese utility weight

Domain	Level	Weight
Occupation	1	0.173
	2	0.166
	3	0.066
	4	0.028
Control over daily life	1	0.147
	2	0.133
	3	0.032
	4	0.009
Looking after yourself	1	0.163
	2	0.155
	3	0.049
	4	0.020
Personal safety	1	0.131
	2	0.111
	3	0.046
	4	0.012
Social participation and involvement	1	0.121
	2	0.103
	3	0.076
	4	0.011
Space and time to be yourself	1	0.205
	2	0.194
	3	0.063
	4	0.000
Feeling supported and encouraged	1	0.130
	2	0.117
	3	0.064
	4	0.011

**Fig. 3:**
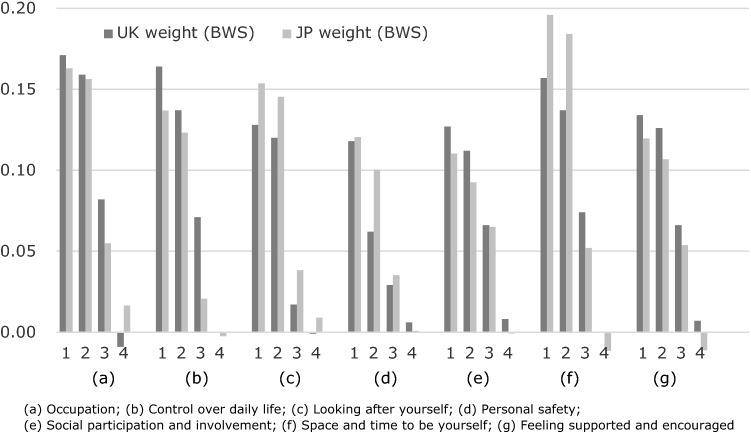
Comparison between Japanese and UK weights

Figure 3 shows a comparison between the weights of the UK and the Japanese. The most preferred item was Level 1 in the “space and time to be yourself.” Respondents showed a strong preference for the “Occupation” and “Looking after yourself” domains. In the UK, the most preferred item was Level 1 in the “occupation” domain. In Japan, the least preferred items were level 4 in the “space and time to be yourself” and “control over daily life” domains. In the UK weighting, the fourth level of the “control over daily life” item was the least preferred. The weights of level 3 in the “control over daily life” domains differed greatly between the Japanese and UK weights.

## Discussion

In this study, we had data from more than 1000 respondents for the BWS tasks and could estimate the BWS coefficients of each ASCOT-Carer state. Using TTO data from about 200 respondents, the BWS coefficients were converted to preference weights, which enabled us to calculate the utility values of each ASCOT-Carer state. No inconsistency of utility weight was found, which means that the values of the levels within each item of the ASCOT-Carer monotonically increased. This means that Japanese respondents could distinguish the difference between each level in the items. The difference in utility weight between level 2 and level 3 tends to be larger than that of the other gaps. The largest one is 0.131 in the “space and time to be yourself.” It seems that the Japanese differences between Level 2 and Level 3 are larger than the UK differences. This may cause the translation of Japanese ASCOT-carers, the preferences of Japanese respondents, or both of them. In addition, these monotonical relations of levels in the items suggest that web surveys using BWS methods are reliable.

The TTO value of the worst ASCOT-Carer state was 0.011. The TTO values and the BWS latent values have linear relationships. The correlation coefficient was 0.96. This justifies the conversion of the BWS value to utility scores by a linear relation with TTO values. In the UK weighting, no TTO survey was performed, and the BWS coefficients were converted to preference weights by assuming that the worst ASCOT-Carer state was 0. In contrast, Japanese weights were determined by mapping the BWS scores to TTO values. Although the anchoring methods were different, similar TTO values were observed. However, it is important that this should be confirmed by the empirical data because the “worst state = 0” setting is based on an assumption.

The Japanese preference weights for ‘looking after yourself’ and ‘space and time to be yourself’ were higher than those of the UK weights for the same domains. When our survey was performed, the outbreak of COVID-19 continued to differ from the time of the UK survey. People had to spend more time in their homes by lockdown, which may have influenced the difference. In Japan, the least preferred item is the fourth level of “space and time to be yourself.” In the UK, the fourth level of “control over daily life” was the least preferred item. This might reflect the differences in the general population’s caregiving preferences between the two countries. In the case of ASCOT SCT4, “the weights of level 3 in “control over daily life” and “occupation” domains greatly higher in Japan than the UK.” [10] This tendency was also observed for the ASCOT-Carer weight.

The TTO value of the worst ASCOT SCT4 [4444444] was − 0.327 [10]. Compared with this value, the TTO value of the worst ASCOT-Carer (0.011) was higher. Similarly, the estimated worst utility value of ASCOT SCT4 was − 0.38, while the worst utility value of ASCOT-Carer was much higher. The frameworks of the survey and statistical method were similar in the two surveys, except difference in the perspective and the BWS survey method (SCT4: face-to-face, Carer: web-based). We cannot know the precise reason for the difference, but the consequence is that people find the worst state of caregivers preferable to that of the care receivers. This result implies that this state is more tolerable if respondents are caregivers than care receivers. This might be caused by respondents’ assumptions: for example, caregivers’ health states were much better than care receivers, caregiving does not continue forever, and caregivers cannot trade their life years because they need to continue caregiving.

One of the most important limitations of this study was the sampling method. TTO scores were collected from one sample to anchor the latent BWS utility collected from a different sample. For the BWS survey, we gathered a quota sample from a web panel owned by the survey company. Web-based surveys are generally less reliable than face-to-face surveys, although the background of respondents is similar to that of the general population. In addition, in the TTO population, we collected respondents from the panel using quota sampling. However, the respondents were limited to those who could visit the survey center in Tokyo. The TTO population tends to include more full-time workers and high-income respondents. Regarding the statistical method, we used a mixed logit model for BWS data, although the UK analysis used the scale heterogeneity MNL (S-MNL) model to “control for differences in error variance in subgroups” [15]. The reason we selected MIXL was as follows: (i) the influence of considering the scale parameter was small, as shown in Nguyen et al. [21]; (ii) our background factor was comparable with the population norms; (iii) statistical analysis of the valuation surveys for PBMs does not generally adjust the background factors such as EQ-5D-5L, EQ-5D-Y, and SF-6Dv2; and (iv) the process of constructing the model was too complicated as shown in Table 1 of Nguyen et al. [21].

Our results can be used to calculate informal caregivers’ utility values and their QALYs. This is the first PBM for caregivers for which Japanese preference weights have been developed. From the perspective of economic evaluation, it is important to reflect caregivers’ QoL when evaluating healthcare technologies. At present, most evaluations neglect caregiver QoL. The estimated utility values from the weights can support the measurement of caregivers’ QoL and can be used for cost-effectiveness analysis.

## Supplementary Information

Below is the link to the electronic supplementary material.

Supplementary file1 (DOCX 903 KB)

## Declarations

### Availability of data and material

The data are available only to the authors because we obtained IC from the respondents under that assurance.
