# Supplementary material for: Japanese preference weights of the Adult Social Care Outcomes Toolkit for Carers (ASCOT-Carer)
Source: Qual Life Res. 2022 Jan 12;31(7):2143–51. doi: 10.1007/s11136-021-03076-w (PMC8752387; doi:10.1007/s11136-021-03076-w)
Supplement: Supplementary file 1 — Supplementary file1 (DOCX 903 KB) [file 11136_2021_3076_MOESM1_ESM.docx]

Supplementary information

**Japanese preference weights of the Adult Social Care Outcomes Toolkit for Carers (ASCOT-Carer)**

*Quality of life research*, Takeru Shiroiwa, Hiromi Nakamura-Thomas, Mai Yamaguchi , Mie Morikawa, Yoko Moriyama, Takashi Fukuda, Stephen Allan, Juliette Malleya; Center for Outcomes Research and Economic Evaluation for Health (C2H), National Institute of Public Health, t.shiroiwa@gmail.com

**Figure S1** Screenshot of BWS and TTO tasks

**
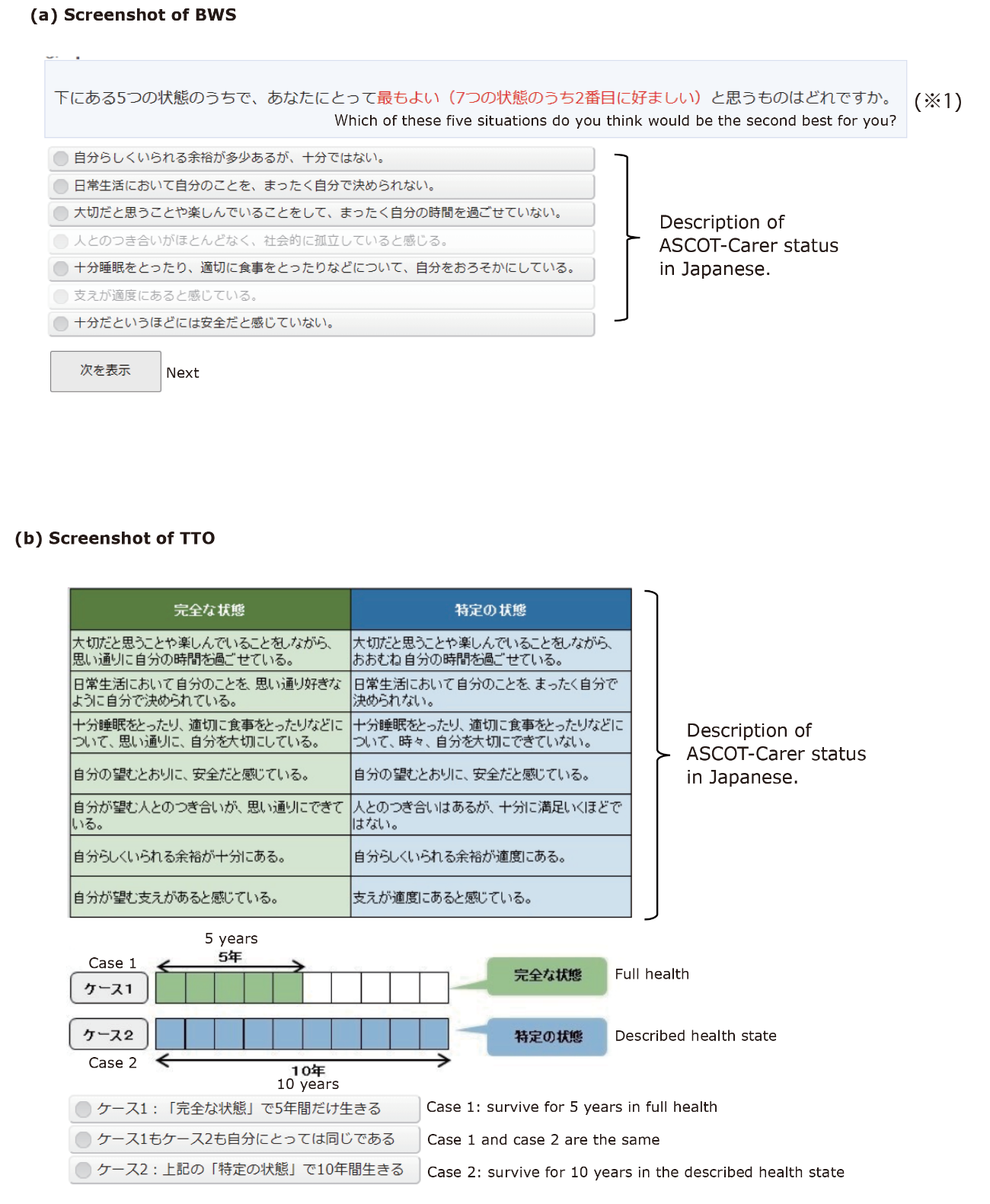
**

**Table S1** Comparison between the final study sample (N=914) and those who were excluded (N=201).

|  | BWS population (N=914) | | Those excluded form BWS (N=220) | |
| --- | --- | --- | --- | --- |
| Gender |  |  |  |  |
| Male | 435 | 47.6% | 122 | 60.7% |
| Female | 479 | 52.4% | 79 | 39.3% |
| Age |  |  |  |  |
| 20–29 | 121 | 13.2% | 63 | 31.3% |
| 30–39 | 130 | 14.2% | 56 | 27.9% |
| 40–49 | 135 | 14.8% | 45 | 22.4% |
| 50–59 | 163 | 17.8% | 20 | 10.0% |
| 60–69 | 180 | 19.7% | 8 | 4.0% |
| 70–79 | 185 | 20.2% | - | - |
| Population of living municipality |  |  |  |  |
| >1,500,000 | 232 | 25.4% | 57 | 28.4% |
| 500,000–1,500,000 | 176 | 19.3% | 39 | 19.4% |
| 200,000–500,000 | 188 | 20.6% | 34 | 16.9% |
| 50,000–200,000 | 217 | 23.7% | 47 | 23.4% |
| <50,000 | 101 | 11.1% | 24 | 11.9% |
| Employment |  |  |  |  |
| Full-time worker | 287 | 31.4% | 119 | 59.2% |
| Part-time worker | 133 | 14.6% | 23 | 11.4% |
| Self-employment | 76 | 8.3% | 14 | 7.0% |
| Retired | 97 | 10.6% | 7 | 3.5% |
| Houseworker | 181 | 19.8% | 15 | 7.5% |
| Student | 32 | 3.5% | 5 | 2.5% |
| Others | 108 | 11.8% | 18 | 9.0% |
| Marital status |  |  |  |  |
| Unmarried | 293 | 32.1% | 112 | 55.7% |
| Married | 536 | 58.6% | 77 | 38.3% |
| Others | 85 | 9.3% | 12 | 6.0% |
| Education |  |  |  |  |
| Elementary or Junior high school | 27 | 3.0% | 6 | 3.0% |
| High school | 275 | 30.1% | 45 | 22.4% |
| College | 182 | 19.9% | 29 | 14.4% |
| University or Graduate | 424 | 46.4% | 120 | 59.7% |
| Others | 6 | 0.7% | 1 | 0.5% |
| Household income (JPY) |  |  |  |  |
| <1 million | 44 | 4.8% | 7 | 3.5% |
| 1 million–2 million | 68 | 7.4% | 13 | 6.5% |
| 2 million–3 million | 94 | 10.3% | 18 | 9.0% |
| 3 million–4 million | 120 | 13.1% | 24 | 11.9% |
| 4 million–5 million | 116 | 12.7% | 17 | 8.5% |
| 5 million–7 million | 133 | 14.6% | 29 | 14.4% |
| 7 million–10 million | 122 | 13.4% | 28 | 13.9% |
| 10 million–15 million | 51 | 5.6% | 27 | 13.4% |
| 15 million–20 million | 12 | 1.3% | 9 | 4.5% |
| >20 million | 7 | 0.8% | 2 | 1.0% |
| Unknown | 147 | 16.1% | 27 | 13.4% |

**Heterogeneity of BWS tasks**

We tested heterogeneity of the best, second Best, worst, second Worst choices. The following table corresponds to Table 4 by Ratcliffe et al. (Soc Sci Med. 2016;157:48-59) for confirming the heterogeneity.

| Hypothesis (H1A) | B = W | 2B = 2W | W = 2W | B = 2B | W = 2B |
| --- | --- | --- | --- | --- | --- |
| best | -12048.6 |  |  | -12048.6 |  |
| worst | -12857.4 |  | -12857.4 |  | -12857.4 |
| 2nd best |  | -10441.9 |  | -10441.9 | -10441.9 |
| 2nd worst |  | -10686.7 | -10686.7 |  |  |
| pool | -25219.7 | -21372.3 | -23612.1 | -22557.1 | -23562.9 |
| chi^2^ (32df) | 313.7 | 243.7 | 68.0 | 66.5 | 263.6 |
| reject H1A | Yes | Yes | Yes | Yes | Yes |

B: Best, W: Worst, 2B: Second best, 2W: Second worst

Following this test, all the null hypotheses were rejected, and heterogeneity of all pairs was shown. However, as our true null hypothesis was, for example, B ≠ W, we suppose it is difficult to simply interpret these results. If this test was applied to the large sample number data, we would wonder whether all the hypotheses (no heterogeneity) would be rejected.

In addition, in such a case, only one choice (maybe B) of data would be used for an analysis similar to that of Ratcliffe et al. Although it is a well-considered method, we are not sure whether it is better to not use the other three choices (W, 2B, and 2W) for analyzing the ”BWS” data.

Based on the comparison of chi^2^ statistics among these pairs, the scores of W = 2W and B = 2B were relatively smaller. Therefore we included all the choices (B, 2B, W and 2W) in the analysis.
